# Characterization of Hispanic, African American, and non‐Hispanic White individuals living in disadvantage neighborhoods: the HABS‐HD Study

**DOI:** 10.1002/alz70860_101015

**Published:** 2025-12-23

**Authors:** Raul Vintimilla, James Hall, Sid E. O'Bryant

**Affiliations:** ^1^ Institute for Translational Research, University of North Texas Health Science Center, Fort Worth, TX, USA

## Abstract

**Background:**

Neighborhood socioeconomic (SE) disadvantage indices are useful tools to measure the impact of social determinants of health. Research have shown that non‐Hispanic Whites (NHW) living in deprived neighborhoods, have better individual SE indicators than Hispanics and African Americans (AA) that live in the same area. Our aim is to characterize a community‐based multiethnic cohort living in disadvantaged neighborhoods.

**Method:**

Data from 2833 individuals (Hispanics 1062, AA 731, NHW 1040) were analyzed. The national level Area Deprivation Index (ADI) was use as a measure of neighborhood SE status. Chi square and ANOVA were utilized to compare demographic, SE, psychological, and health variables between ethnic groups in the whole sample and in those living in the 20% most deprived neighborhoods (ADI deciles 8, 9, and 10). Logistic regression models split by ethnicity were run to determine the impact of living in the most deprived neighborhoods on comorbidities. Age, gender, education, and income were entered as covariates. Statistical significance was set at ≤ 0.05.

**Result:**

In the whole sample, significant differences between ethnicities were found in demographic, SE, psychological, and health variables. When comparing individuals living in the most deprived neighborhoods, Hispanics had less education, lower income, and poorer performance on MMSE than AA and NHW. Hispanics were also less likely to be insured, having a health provider, and reported less social support. Compared to NHW, Hispanics were younger, perform worse in memory measures, and were more likely to be female and having cognitive impairment. When compared to NHW, AA were younger, had lower income, performed worse on MMSE, and were more likely to have cognitive impairment. Among Hispanics, ADI was associated with diabetes (OR = 2.15), obesity (OR = 1.28), and depression (OR = 1.38). In AA, ADI was associated with hypertension (OR = 1.54), diabetes (OR = 1.72), and depression (1.82). NHW living in the most deprived neighborhoods were more likely to have hypertension (OR = 1.81).

**Conclusion:**

Findings revealed that demographic, socioeconomic, psychological, and health characteristics of individuals living in the most deprived neighborhoods are different among the US main ethnic groups.

